# Venom Proteins from Parasitoid Wasps and Their Biological Functions

**DOI:** 10.3390/toxins7072385

**Published:** 2015-06-26

**Authors:** Sébastien J. M. Moreau, Sassan Asgari

**Affiliations:** 1 Institut de Recherche sur la Biologie de l’Insecte, Centre National de la Recherche Scientifique Unité Mixte de Recherche 7261, Université François-Rabelais, Unité de Formation et de Recherche Sciences et Techniques, Parc Grandmont, 37200 Tours, France; 2 School of Biological Sciences, the University of Queensland, Brisbane, QLD 4067, Australia

**Keywords:** venom, endoparasitoid, ectoparasitoids, parasitism, host, wasp

## Abstract

Parasitoid wasps are valuable biological control agents that suppress their host populations. Factors introduced by the female wasp at parasitization play significant roles in facilitating successful development of the parasitoid larva either inside (endoparasitoid) or outside (ectoparasitoid) the host. Wasp venoms consist of a complex cocktail of proteinacious and non-proteinacious components that may offer agrichemicals as well as pharmaceutical components to improve pest management or health related disorders. Undesirably, the constituents of only a small number of wasp venoms are known. In this article, we review the latest research on venom from parasitoid wasps with an emphasis on their biological function, applications and new approaches used in venom studies.

## 1. Biological Functions of Parasitoid Wasp Venoms

Parasitoid wasps belong to the order Hymenoptera and are valuable insects in suppressing host populations either through natural or augmented biological control. Typically, the female wasp deposits its egg inside (endoparasitoid) or outside (ectoparasitoid) the host (mostly arthropods) where the emerged parasitoid larva continues to feed. Eventually, the host dies due to parasitism, although there seem to be examples in which the host may survive and continue to reproduce (e.g., [1]). As a consequence of the differing lifestyle, the physiological requirements and impacts on the host by endoparasitoids and ectoparasitoids may vary [2]. Components injected into the host at the time of parasitization play vital roles in facilitating successful parasitism, including venom and ovarian/calyx fluid. These may or may not contain symbiotic viruses or virus-like particles that contribute to host manipulation, in particular in endoparasitoids.

Similar to venom found in most venomous animals, venom fluid from parasitoid wasps consists of a cocktail of proteinacious and non-proteinacious compounds. While various studies that have focused on determining the venom profile of ecto- and endoparasitoid venoms (see below) have revealed the presence of several conserved proteins between the two parasitic wasp groups, venom appears to serve different purposes in the two groups. In general, venom from ectoparasitoids is largely involved in the host paralysis (short or long-term) to secure feeding of the ectoparasitic larva outside the host, whereas endoparasitoids’ venom rarely causes paralysis but facilitates parasitization by interfering with the host immune system, development or synergizing the effects of other maternal factors introduced into the host (e.g., polydnaviruses, PDVs). In this review, we will discuss the major biological functions of parasitoid wasp venoms, latest approaches used for venom studies and some of the potential applications of venom proteins from those insects.

### 1.1. Ectoparasitoids

The primary function of venom in most ectoparasitoids (in particular when the host is at the active stage, e.g., larval/nymphal stage) is induction of short- to long-term paralysis/lethargy in the host and developmental arrest. However, venom may play other roles in facilitating parasitization, such as suppressing the host immunity (e.g., [3,4]) or interrupting development (e.g., [5,6]). Despite thousands of ectoparasitoids species known, there are only very limited number of venom components identified from a small number of ectoparasitoids.

The parasitoid *Ampulex compressa* injects a cocktail of neurotoxins into the central nervous system of its cockroach prey. This involves two consecutive stings, one in the thorax, which leads to transient paralysis of the front legs due to post-synaptic blockage of central cholinergic synaptic transmission, and a second one by injection of venom specifically inside the sub-esophageal ganglion of its cockroach prey, which induces a 30 min intense grooming in the prey (induced by dopamine) followed by a long-lasting lethargic effect [7,8]. The latter effect is most likely caused by venom affecting the opoid system [9] or octopaminergic receptor [10]. The venom from *A. compressa* contains GABA (inhibitory neurotransmitter) and ß-alanine (GABA receptor agonist), and taurine (impairs the re-update of GABA from the synaptic cleft) [11] (Table 1). It has been suggested that these three main components have both pre- and post-synaptic effects on GABA-gated chloride channels.

Venom from the digger wasp *Philanthus triangulum* contains philanthotoxins which affect both the central and the peripheral nervous system of the prey by presynaptic as well as a postsynaptic blockage of neuromuscular transmission [12,13]. Specifically, the toxins inhibit the re102lease of glutamate and block the post-synaptic glutamate receptors. In addition, δ-philanthotoxin inhibits the nicotinic acetylcholine receptors in the central nervous system [14]. *Bracon hebetor* is another ectoparasitoid with a potent venom causing host paralysis [15]. Three proteins with molecular masses of about 73 kDa were found in the venom, two of them (Brh-I and -II) being insecticidal when injected into lepidopteran larvae. Of the two, Brh-I was found to be more toxic against the larvae of the cotton bollworm, *Heliothis virescens*. *Liris niger*, which hunts, paralyses and parasitizes the mole cricket, injects venom into the nervous system leading to blockage of voltage-gated sodium inward currents, and synaptic transmission [16]. The constituents of the venom, which comprise of proteins from 3.4–200 kDa have not been well characterized [17]. The ectoparasitoid *Eupelmus orientalis* venom causes permanent host paralysis and developmental arrest, the two effects found to be independent of each other [18]. In the venom, hyaluronidase and phospholipase activities were detected.

**Table 1:** Major biological functions of venom from parasitoid wasps.

Biological Functions	Wasp	Parasitism	Host	Reference
*Paralysis*				
pimplin	*Pimpla hypochondriaca*	Endo	*Lacanobia oleracea*	[19]
philanthotoxins	*Philanthus triangulum*	Ecto	*Schistocerca gregaria*	[12]
Brh-I & -II	*Bracon hebetor*	Ecto	*Diaprepes abbreviatus*	[20]
GABA, β-alanine, taurine	*Ampulex compressa*	Ecto	*Periplaneta americana*	[11]
*Hemocyte inactivation*				
VPr1	*Pimpla hypochondriaca*	Endo	*L. oleracea*	[21]
VPr3	*Pimpla hypochondriaca*	Endo	*L. oleracea*	[22]
Vn.11	*Pteromalus puparum*	Endo	*Pieris rapae*	[23]
VP P4, RhoGAP	*Leptopilina boulardi*	Endo	*Drosophila melanogaster*	[24]
calreticulin	*Cotesia rubecula*	Endo	*P. rapae*	[25]
	*Pteromalus puparum*	Endo	*P. rapae*	[26]
SERCA *	*Ganaspis* sp.1	Endo	*D. melanogaster*	[27]
*Inhibition of melanization*				
LbSPNy	*Leptopilina boulardi*	Endo	*D. melanogaster*	[28]
Vn50	*Cotesia rubecula*	Endo	*P. rapae*	[29]
*Interrupting development*				
Reprolysin	*Eulophus pennicornis*	Ecto	*L. oleracea*	[6]
*Enhancing PDVs*				
Vn1.5	*Cotesia rubecula*	Endo	*P. rapae*	[30]
*Castration*				
γ-glutamyl transpeptidase	*Aphidius ervi*	Endo	*Acyrthosiphon pisum*	[31]
*Anti-microbial*				
PP13, PP102, PP113	*Pteromalus puparum*	Endo	*P. rapae*	[32]

* sarco/endoplasmic reticulum calcium ATPase.

*Nasonia vitripennis* is a model ectoparasitoid wasp with its genome completely sequenced. It parasitizes the pupal stage of a number of fly species as its host. The wasp’s venom inflicts a variety of effects on the host including developmental arrest and decrease in metabolism and immunity [33,34,35,36]. Using a suppression subtractive hybridization method, it was shown that the venom from *N. vitripennis* caused differential gene expression in the hemocytes of the host pupae *Musca domestica* [37]. At 1 h after venom application, 133 expressed sequence tags (ESTs) showed decrease in transcript levels and 111 ESTs were found to be upregulated. The altered genes were mostly related to various biological functions such as immunity, apoptosis, stress response, metabolism and regulation of transcription/translation. The outcome shows a profound impact of venom injection on the host hemocytes. In another study, the global effects of *N. vitripennis* on an alternative fly host, *Sarcophaga bullata*, were studied using high throughput RNA sequencing (RNA-seq) of the whole host body following venom treatment [38]. Overall, about 147 host genes were significantly differentially expressed due to envenomation with the percentage of differentially expressed genes increasing as the parasitization progressed. The genes were mostly related to chitin metabolism, cell death, immunity and metabolism. In a similar study, it was found that parasitization of *Sarcophaga crassipalpis* pupa by *N. vitripennis* led to differential expression of only one gene at 3 h after parasitization but 128 genes at 25 h post-parasitization [39]. Similarly, these genes were involved in metabolism, development, immune responses and apoptosis. While various proteins have been identified in *N. vitripennis* venom such as serpins, laccases, metalloproteases, calreticulin, chitinase and serine proteases, their functions in alterations of host physiology have mainly been implied rather than experimentally tested (reviewed in [36]).

In a different pupal ectoparasitoid, *Scleroderma guani*, the transcriptome of the host *Tenebrio molitor* was shown to change following envenomation and the differentially expressed genes were related to similar categories changed in *N. vitripennis* hosts [40]. This indicates that parasitoids manipulate similar genes and pathways that facilitate parasitization.

### 1.2. Endoparasitoids

In endoparasitoids, venom usually does not have a paralytic effect on the host, except in a few examples in which transient paralysis has been recorded [41,42,43,44]. The host normally recovers in a few minutes or within one hour after parasitization. By adopting a koinobiont life style, which allows further development of the host, and living inside the host, endoparasitoids do not require inducing a long-term paralysis in the host. However, the presence of toxin-like peptides in their venom strengthens the assumption that they shared a common ancestor with ectoparasitoids [45].

Deposition of the endoparasitoid egg inside the host exposes the developing parasitoid to host immune responses, mostly encapsulation, which is engulfing the egg/larva with multi-layers of hemocytes. This response is often accompanied by melanization, a cascade of proteolytic reactions leading to the deposition of melanin and production of phenolic intermediates [2]. In addition, as most endoparasitoids allow further development of their host while their juvenile stage is feeding inside the host, regulation of the host development and metabolism is essential. Components introduced into the host at parasitization play the main part in conditioning the host physiology to facilitate endoparasitoid development. While all endoparasitoids inject venom at parasitization, it may not be sufficient to subvert the host physiology. In a large number of parasitoid-host systems injection of supplementary proteins produced in the calyx region of the ovaries or viruses that replicate in the ovaries or venom glands are essential to guarantee successful parasitism.

In many instances, venom is the sole maternal factor that accompanies the endoparasitoid egg, which is sufficient to facilitate parasitization. A well-studied example is *Pimpla hypochondriaca.* The venom from *P. hypochondriaca* consists of several enzymes, protease inhibitors, neurotoxin-like factors and anti-hemocyte aggregation compounds (Table 1). While the function of most of these compounds remains unexplored, evidence suggests that they could be involved in venom homeostasis [46], transient paralysis [19], cytotoxicity [47], and inactivation of hemocytes [22,47]. In *Leptopilina boulardi*, that parasitizes *Drosophila* species, venom is essential to suppress the encapsulation response [24]. The major protein involved is a RhoGAP (Rac GTPase Activating protein) that affects the spreading and aggregation of lamellocytes rendering them incapable of forming a capsule [48]. This might be achieved by targeting two *Drosophila* Rho GTPases, Rac1 and Rac2, essential for encapsulation of parasitoid eggs, after entering the host hemocytes [49]. Venom from *Pteromalus puparum*, and in particular a 24.1 kDa protein (Vn.11), affects the host hemocytes causing reduction in total hemocyte count and their ability to encapsulate foreign objects [23,50]. Sequencing of forward subtractive libraries of *Pieris rapae* hemocytes and fat body after *P. puparum* venom injection showed that the expression levels of a large number of genes were significantly altered (113 in hemocytes and 221 genes in fat body down-regulated) [51]. Many of the identified genes were immune related, as well some that were in non-immune categories. Consistently, a C-type lectin was found down-regulated affecting the activation of the host immune system [52]. In addition to immune suppression, venom from *P. puparum* also affects host development by inducing endocrine changes in the host [53]. Accordingly, juvenile hormone (JH) titers were significantly higher, and JH esterase and ecdysteroid titers significantly lower in parasitized or venom-injected *P. rapae* larvae as compared to control non-treated larvae. These changes would ensure that the larvae have a prolonged larval period. Venom from *Aphidius ervi* causes cell death in the ovarial tissues of the host *Acyrthosiphon pisum* leading to host castration [54]. The apoptotic effect is presumably caused by a γ-glutamyl transpeptidase in the venom fluid [31].

In endoparasitoids that produce viruses or virus-like particles (VLPs), venom functions vary in different host-parasitoid systems ranging from no effect to having overlapping functions with genes expressed from the encapsidated genes within the VLPs or synergise their function. Venom from *Tranosema rostrale* had no effect on host alterations (reduction in total hemocyte count and inhibition of melanization) observed in natural parasitization or when calyx fluid alone was injected [55]. This implied that venom might not play a significant role in parasitization. Similarly, despite having a complex mixture of proteins, venom from *Hyposoter didymator* was found not required for successful parasitism [56]. In addition, in a number of other ichneumonid wasps with PDVs, venom has been found not essential for successful parasitism [57,58,59,60].

On the other hand, in a number of host-parasitoid systems venom is essential for proper function of PDVs. PDVs are virus-like particles that are produced in the calyx region of a large number of wasps from Ichneumonidae and Braconidae [61]. They are defective viruses in that they are not able to replicate independent of the parasitoid since the replication machinery (related to nudiviruses) is integrated into the wasp genome [62,63]. For this, they only replicate in the wasp ovaries and not in the parasitoid’s host following parasitization. The genes encapsidated in the particles, which appear to be mostly of insect origin, are expressed in the host interfering with the host physiology, in particular suppressing the immune system [64].

Venom has been found to synergize the effect of PDVs. For instance, venom enhances the effects of *Microplitis demolitor* PDVs on host hemocytes (inhibition of cell spreading) in a dose-dependent manner [65] and in delaying development [66]. In *Cotesia melanoscela*, venom is required for entry and unpackaging of PDVs [67], and in *Cotesia rubecula*, in the absence of venom, PDV genes were not expressed in hemocytes [30]. A 1.5 kDa venom peptide (Vn1.5) was found to facilitate expression of CrPDV genes. In *Cotesia nigriceps* both venom and calyx fluid were needed to cause cessation of growth in *Heliothis virescens* larvae [68]. In addition to enhancing PDV functions, venom proteins from endoparasitoids may affect host immunity as well as development. For example, a number of venom proteins interfere with the proper function of host hemocytes. In *C. rubecula*, a calreticulin was shown to inhibit *P. rapae* hemocyte spreading debilitating them from the encapsulation response [25]. Calreticulin from *P. puparum* venom was also found to inhibit *P. rapae* hemocyte spreading and encapsulation response [26]. Calreticulin has been reported from the venom of other endoparasitoids (e.g., [69]) as well as ectoparasitoids (e.g., [70]). A sarco/endoplasmic reticulum calcium ATPase (SERCA) pump protein from a less known parasitoid of *D. melanogaster*, *Ganaspis* sp. 1, was shown to inhibit the activation of plasmatocytes by suppressing calcium burst required for their activation [27]. As a consequence, hemocytes failed to carry out encapsulation. In another parasitoid of *D. melanogaster*, *Asobara japonica*, venom suppressed hemocyte functions but had no effect on humoral responses [71].

Apart from the effects of venom on cellular immunity, the humoral (non-cellular) arm of the host immune system could be a target of venom proteins. For instance, inhibition of host hemolymph melanization is usually a consequence of parasitization in which venom proteins may play a role. A 50 kDa protein (Vn50) from *C. rubecula* was found to inhibit the activation of prophenoloxidase (proPO) to phenoloxidase (PO), a key enzyme in the melanization pathway [72]. This is due to structural resemblance of Vn50 to serine protease homologs (SPHs) [73], which normally facilitate activation of the enzyme by proPO activating protein (PAP) [74], and competitive binding to proPO and PAP [75]. Venom from *P. puparum* reduced transcription of antimicrobial peptides such as cecropin, lysozyme, attacin, lebocin, proline-rich AMP, *etc*. in hemocytes and fat body of *P. rapae* larvae [51]. In addition, transcript levels of genes involved in proPO activation cascade, such as PAPs, were down-regulated.

In addition to suppressing the host immune system, interfering with host development could be another function of venom from some endoparasitoids. A 66 kDa venom protein from *Cardiochiles nigriceps* in combination with calyx fluid was found responsible for delaying larval development and inhibit pupation in *H. virescens* larvae [76]. Calyx fluid alone was not able to induce the same effect in the host. This effect appears to be due to degradation of the prothoracic glands [68,77].

## 2. New Approaches in Venom Studies

### 2.1. RNAi

RNA interference is an ancient and conserved response to the presence of double stranded RNA (dsRNA) in eukaryotic cells [78]. The source of dsRNA might be exogenous or endogenous. Exogenous dsRNA could be viral RNA genome, viral replicative intermediates, overlapping viral transcripts produced during viral replication (mostly in DNA viruses), or *in vitro* synthesized dsRNA. Once the presence of dsRNA is sensed in the cell, a ribonuclease enzyme called Dicer, cleaves the dsRNA into short interfering RNAs (siRNAs). siRNAs induce formation of the RNA Induced Silencing Complex (RISC) in which an argonaute (Ago) protein plays a major role. siRNA-loaded RISC complex facilitates binding of the siRNAs to their complementary target sequences and their subsequent cleavage. Transfection of dsRNA/siRNAs into cells or whole organisms are routinely used to knockdown transcript levels of target genes. In insects, the level of success in application of dsRNA for gene silencing studies has been variable; working really well in some insects and not in others [79]. Given knocking out genes is not possible in many non-model insects, gene silencing by RNAi using long dsRNA or siRNA has been quite useful.

Application of RNAi could also be useful in studying the function of specific venom proteins in host-parasitoid interaction. In a recent study, Colinet *et al.* utilized RNAi to successfully silence the *RhoGap* gene abundant in *L. boulardi* venom [80]. Silencing was achieved by microinjection of dsRNA specific to the gene into the parasitoid pupae. The results showed near complete silencing of the gene and lack of the protein detection in the venom reservoir of the wasps emerged from gene-specific dsRNA injected pupae. Interestingly, the silencing effect remained stable throughout the entire wasps’ lifetime. This initial step towards demonstration of successful silencing of a gene coding for a venom protein provided a new experimental tool in investigating the specific role of the proteins in host-parasitoid interactions and their importance in the success of parasitism.

### 2.2. High Throughput Methods: Transcriptomic, Proteomics, Peptidomics

Major advances and cost reductions in high throughput analyses of RNA and proteins have provided opportunities for researchers to identify and gain a better understanding of the diversity of venom proteins/peptides from various animals. In addition, these approaches could enable analysis of venom impacts on the host transcriptome in more depth. These studies in general show the presence of conserved proteins present in venom from endo- and ectoparasitoids but also some that are unique to each parasitoid. Further, a large number of proteins/peptides are being discovered with no significant similarity to other proteins with known functions.

In 2010, Zhu *et al.* performed a proteomic analysis of the venom from the endoparasitoid *P. puparum* which allowed identifying 12 out of 56 soluble proteins extracted from a venom apparatus homogenate. While a number of proteins highly similar to venom proteins identified in other hymenopteran species were found (e.g., venom acid phosphatase, calreticulin), the method used did not allow identification of other possible venom components among proteins of cellular origins with certainty.

In the same year, the first exhaustive identification of venom components of a parasitoid wasp was carried out using a combination of sequencing ESTs from a venom gland library and nano-LC-MS/MS analysis of peptides from pure venom isolated from the venom reservoir of the egg-larval parasitoid *Chelonus inanitus* [81]. The main venom components were a number of enzymes (chitinase, esterase, metalloprotease-like, C1A protease, serine protease), mucin-like peritrophins, lectin-like proteins and yellow-e3 like venom protein similar to *Apis mellifera* protein produced in the head and hypopharyngeal gland of honeybee workers. A number of proteins were also found to be unique to the parasitoid. In a complementary work, it was shown that the venom proteins enhance PDV entry into the host cells and facilitate placement of the parasitoid egg in the host embryo’s hemocoel [82].

Using a combined transcriptomic and proteomic approach, Colinet *et al.* found 16 putative venom proteins from *A. ervi* [83]. The most abundant proteins were three γ-glutamyl transpeptidases (γ-GTs), two of which are likely coded by alleles of the same gene and the third one unrelated to the other two and most likely inactive due to a mutation in the active site. The product of one of the two alleles was previously shown to cause castration in the host aphid *A. pisum* by causing apoptosis and subsequent tissue degradation in the ovaries [31,54]. The study also resulted in the identification of proteins present in other parasitoid venoms such as SPHs, neprilysin-like and cysteine-rich toxins; and some being unique to the parasitoid; such as endoplasmin [83].

In another transcriptomic study, a large number of unigenes were identified from the venom of *Leptopilina heterotoma*, an endoparasitoid of *D. melanogaster* [84]. Similar to other such studies, several venom proteins were identified that were conserved among endo- and ectoparasitoids, as well as several unique genes. The components of venom from *L. heterotoma*, including VLPs, are responsible for suppression of the host immune system and delay in the host larval development [85,86]. The exact impact of venom *versus* VLPs in the effects observed in the host is not clear. In a different study, the venom composition of *L. heterotoma* and *L. boulardi*, two parasitoids of *D. melanogaster* with different parasitism strategies were explored using a combination of RNA-seq and proteomics approaches. *L. boulardi* is specialized on *D. melanogaster* and inhibits cellular immunity by inhibiting hemocytes from encapsulation, while *L. heterotoma* parasitizes different species of *Drosophila* and causes destruction of the host hemocytes [87]. This study led to the identification of 129 and 176 proteins in *L. boulardi* and *L. heterotoma* venoms, respectively. A large number of proteins were found in venom from both species but also some that were unique to each which presumably may contribute towards their different strategies in parasitism [69].

Following the availability of the complete genome of the ectoparasitoid wasp *Nasonia vitripennis*, de Graaf *et al.* used a combination of bioinformatics and proteomic analyses to determine the profile of proteins present in the wasp’s venom reservoir [70]. The bioinformatics approach was based on digging into the genome sequences using similarity with other known venom proteins. The proteomics approach was based on using two mass spectrometry approaches: off-line 2D liquid chromatography matrix-assisted laser desorption/ionization time-of-flight (2D-LC-MALDI-TOF) MS and a 2D liquid chromatography electrospray ionization Founer transform ion cyclotron resonance (2D-LC-ESI-FT-ICR). The outcome was identification of 79 proteins among which half of them were proteins that were either unknown or not yet associated to insect venoms. The major groups were proteases/peptidases, protease inhibitors, enzymes involved in carbohydrate, DNA and glutathione metabolism, esterases, recognition proteins, and immune related. Serine proteases and protease inhibitors were overrepresented in the venom fluid [70].

Similarly, a combination of transcriptome sequencing and proteomics was used to identify the protein profile of venom proteins in *M. demolitor* [64]. This study demonstrated the presence of several venom proteins found in other parasitoids (e.g. a reprolysin-like metalloprotease and Ci-48a), but also some unique hypothetical proteins. This study presented further evidence of recruitment of insect proteins into venom by gene duplication and modification. Further, comparison of *M. demolitor* PDV gene products and venom proteins showed no overlap suggesting separate functions of the products.

## 3. Venom Protein Evolution and Diversity

### 3.1. Venom Diversity within the Hymenoptera: Who Are the Outliers?

Hymenoptera of the suborder Apocrita would gather more than 300,000 species, representing 10% to 20% of all insect species currently living on earth [2,88,89]. The suborder Apocrita is divided into two major groups, Parasitica, that possess an ovipositor (terebra or drill) functioning as a dual egg-laying and venom injecting organ, and Aculeata, in which the ancestral ovipositor has been fully modified for injection of venom (aculeus or sting) [90,91]. All modern Apocrita share a common ancestral parasitic origin [92] from which they have successfully evolved to display diversified lifestyles and nutritional behaviours, including parasitism of plants or arthropods, predation, phytophagy and omnivory. Depending on the species, venoms are being used as defensive agents against predators, competitors and pathogens, hunting weapons, manipulators of host physiology, repellents and trail, alarm, sex, recognition, aggregation and attractant-recruitment pheromones [93,94]. Available data on the composition of Apocrita venoms are highly heterogeneous depending on the considered superfamilies, and the extent of the complexity and diversity of these arsenals is still difficult to estimate with precision.

During the past 50 years, proteins and peptides have focused most of the attention of investigators interested in the composition of Hymenoptera venoms. Around 70 Hymenoptera species (out of 300,000 venom-producing species!) were studied to this aim. In the Vespoidea superfamily, which gathers ants and solitary and social wasps, an overall of 138 different proteins and peptides out of 43 species investigated were characterized. In Chalcidoidea, Apoidea and Ichneumonoidea, a lower number of species have been studied (3, 6 and 11 respectively) with lead species deeply investigated through venomic approaches (e.g., *N. vitripennis*, *A. mellifera*, *C. inanitus*, *M. demolitor*). In Cynipoidea, investigations were only performed on three parasitoids of *Drosophila* belonging to the genus *Leptopilina* or to the genus *Ganaspis*. In the seven remaining superfamilies of Hymenoptera, which represent an estimated number of more than 20,000 species [2], there is simply no data available. This underlines how far we are from grasping the richness and diversity of Hymenoptera venoms even for the most studied groups of species. It stresses too how extensive venomic studies can quantitatively and qualitatively improve our knowledge of this molecular diversity. An overview of main families of proteins and peptides characterized until now from Hymenoptera venoms can be found in Table 1.

Most parasitoid species have evolved under strong selective pressures and have adapted to a restricted range of hosts thank to specific strategies of virulence in which venoms can play a predominant role [95]. The very first analytical works led on parasitoid venoms from the late eighties intended to compare their composition to that of social hymenopteran species which were, by far, the best known at this period. These pioneer studies suggested that unlike venoms from social Aculeates, parasitoid venoms lacked small proteins and peptides and could be characterized instead by the presence of large venom proteins whose molecular masses frequently exceeded 100 kDa [90,96,97]. This statement was taken up during the following two decades and seemed to be confirmed for a while by the description of large proteins in venoms of parasitoids [15,18,19,72,98,99,100,101,102]. In parallel, however, an increasing number of peptides and small proteins of molecular masses lower than 15 kDa have also been discovered in the venoms of parasitoid wasps belonging to distant families such as Eupelmidae, Pteromalidae, Braconidae or Ichneumonidae [18,19,30,32,41,72,81,101,103,104]. In fact, there is such a diversity of functions represented among proteins that were identified to date in parasitoid venoms, that presence of proteins of high molecular masses is definitely of second importance and cannot reasonably be hold as a common distinctive feature of parasitoids’ venoms.

Incidentally, if their functional diversity was more explored and taken into consideration, this could put an end to the temptation to see in the venom of social aculeate Hymenoptera a classical pattern for all hymenopteran venoms and to consider parasitoids as outliers. At most it may be considered now that venoms of social species, which only gather a fraction of the most recent species of the order and which have independently evolved from several parasitoid lineages [2,89], constitute rather an exception than a paradigm. Indeed, these venoms are particularly rich in neurotoxic, cytolytic and antimicrobial peptides that fulfill key roles in capture and conservation of preys, defense against competitors and prevention of brood nest contamination by microorganisms [105]. The presence and abundance of such compounds make sense only with respect to particular lifestyles (eusocial, subsocial or solitary life) and feeding modes (omnivory, phytophagy, predation) and have certainly played an important role in the diversification of these species and their ecological dominance [89]. But they have little in common with venom compounds used as virulence factors needed to finely adjust and manipulate the internal physiological balance of hosts, a constraint experienced by most parasitic wasps, and endoparasitoid species in particular.

This confusion mainly originates from a widespread anthropocentric view in addressing the issue of venom, that some extensive works such as “Venoms of the Hymenoptera” [90] and other recent papers [2,106,107] have not totally succeeded to clear up. According to this conception, the most important venoms to man would naturally constitute the most important venoms. One may understand that this outdated view has served as a primitive conceptual matrix to the pioneer comparative works of the eighties because they preceded the functional examination of parasitoid venoms. It is a bit more surprising how often this view is fostered in more recent comparative papers (see for instance [108,109]) aiming at underlying the potential of venomic approaches for pharmaceutical discovery, but in which the composition richness and subtlety of functions displayed by the venoms of parasitic wasps and other Hymenoptera are simply ignored to the benefit of a lapidary mention of the “predatory” and “defensive” roles of the venoms from “ants, wasps and bees”. It does not only deny hymenopteran venom diversity, it also neglects venom variability and complexity, its inter- and intra-individual corollary dimensions. To go further and break with “an anthropocentric view of toxicity”, Fry *et al.* (2009) [110] have proposed a global definition regarding “(…) venom as a secretion, produced in a specialized gland in one animal, and delivered to a target animal through the infliction of a wound regardless of how tiny it could be, which contains molecules that disrupt normal physiological or biochemical processes so as to facilitate feeding or defense by the producing animal”. With a little effort this interesting definition could have been useful, but it excludes important cases, like the possibility for venom to be injected into a host plant and not just into an animal. The case is frequent for instance in Cynipidae which develop as parasites of wild roses or oaks and which inject venom in host plant tissues during oviposition [111]. The oak gall wasp *Biorhiza pallida* even possesses one of the largest venom apparatuses found in a hymenopteran in proportion of the body size [112]. The exact functions of *B. pallida*’s venom are not known to date as those of hundreds of thousands of other parasitic wasps associated to animal or plant hosts. It may thus be also hazardous to define what is or not venom by reference to a restrictive set of functions, such as feeding and defense, because we still largely ignore all what venoms can achieve. The proposed definition also discards the cases in which venoms can act on another organism in the absence of wounding, for instance through venom spraying for prophylactic or defensive purposes and venom deposition in order to serve as a pheromone [105].

It is noteworthy that little is known, in Hymenoptera, on small size venom components belonging to other biochemical classes than proteins and peptides. Knowledge acquired in this domain only comes from the study of some social species belonging to Apoidea and Vespoidea superfamilies. A small set of biologically active amines either acting as smooth muscle agonists, pain-inducing or paralytic factors have been described in venoms of solitary or social Aculeates [11,90]. They notably include histamine, acetylcholine, 5-hydroxytryptamine, bradykinin, GABA, taurine, β-alanine, serotonine, tyramine, dopamine, noradrenaline and adrenaline. Formic acid is the most famous ant venom component and to date, the single organic acid known from Hymenoptera venoms. Some ant venoms may also contain a diversified range of alkaloids, monoterpene hydrocarbons, aromatic nitrogen-containing compounds and lactones [93]. New available techniques for metabolomics analyses could be useful to investigate the presence of such molecules in venoms of other Hymenoptera superfamilies and may even reveal other unexplored classes of active venom metabolites (e.g., free amino acids, lipids, polysaccharides, sterols, *etc.*).

In summary, by highlighting papers of particular interest that focus on the main venomous functions of species of importance, one may sometimes be at risk of simply missing the essential aspects of an issue.

### 3.2. Factors Shaping Venom Complexity in Parasitoid Species

A given parasitoid species is supposed to obtain several adaptive advantages from the production of a complex venom [99]: (1) the combined actions of different venom components allow targeting of several host functions either simultaneously or sequentially; (2) the effects of the venom components may be complementary or even cumulative; (3) the likelihood that hosts simultaneously develop resistances against multiple venom components is low. On the other hand, the production of venom, which may start before adult emergence [97], is reputed to be costly. Biochemical, proteomic and transcriptomic analyses on parasitoid venoms and venom glands have shown a long time ago that these tissues generally express a small number of highly abundant proteins and peptides and a large number of low abundance products [27,64,70,80,81,96,113]. This raises at least two questions: First, why do investigators continue to expend public funds into costly high-throughput transcriptomic methods that will generate millions of redundant sequence reads to identify only a few dozen major venom proteins? Second, what allows a secreted product to be selected among these few dozen key components? There are a whole bunch of politically incorrect answers to the first question which might be easily found elsewhere, suffice to say that some like to be exhaustive at someone else’s expense, and that deep sequencing methods are appropriate tools to address the issue of inter- and intraspecific venom variability [114].

Concerning the second point, Fry *et al.* (2009) [110,115] have noticed a high proportion of convergently recruited protein families among secretions of a wide range of venomous organisms, suggesting that similar structural and/or functional constraints could influence toxins recruitment across the animal kingdom. In parasitoids, venom complexity is the result of a balance between benefits and costs that seems to have favored the selection and production in abundance of a restricted number of venom proteins that are congruent with strict requirements of safety towards the producing organism and efficiency towards targets. As in other venomous animals, potentialities for recruitment and evolution of parasitoid venom proteins greatly depend on individual, populational and ecological factors.

#### 3.2.1. Individual Factors

Individual factors gather physiological features that may affect venom complexity. They may include specific biochemical properties such as the acidic nature of the venomous secretions, the histological organization of venom glands in a simple glandular epithelium and the occasional presence of structures like an internal chitin layer in the secretory duct and the reservoir of the venom apparatus [116]. The tissue organization could have greatly influenced the recruitment of compatible compounds, selected under the double necessity to be devoid of any autotoxic effect and to remain (or to only become) bioactive upon injection. The recruitment of new venom proteins is supposedly mediated, in parasitic wasps, by gene duplication events eventually followed by changes in protein addressing and processing steps [41,114]. Prevention of autotoxicity and conservation of bioactivity may be achieved by additional changes in substrate specificity or catalytic sites of the venom enzymes by comparison to their cellular homologs [72,80,101,114,117]. It can also rely on the presence in the venom of specific enzyme inhibitors [118] and molecular chaperones [119] or secretion of venom enzymes as inactive precursors [41].

In most parasitoid species studied to date, venoms were either reported to constitute the main predominant factors of virulence or to facilitate the action of other factors [30,82,116,120,121,122,123]. It is worthy to underline that in the former case, numerous studies have focused on the identification of prime venom components while paying little attention to other secondary venom molecules capable of potentiating or regulating their action. When venoms act synergistically with other factors of virulence (*i.e.*, symbiotic PDVs, VLPs, ovarian fluids, larval secretions, *etc.*), the molecular basis underlying facilitating processes remain largely unknown, with few exceptions [30]. Even more intriguing are parasitoid venoms that were acknowledged to be non-essential for the survival of the parasitoid wasps’ progeny. This is notably the case for the venoms of the braconid *Cotesia congregata* [124] and the ichneumonid *C. sonorensis* [60], *Tranosema rostrale* [125] and *H. didymator* [56]. Subtle interactions are hence likely to take place (1) between venom components; (2) between venom and other factors of virulence; and (3) between venom and various targets. These interactions probably weighted significantly on venom complexity. Remarkably, functional redundancy seems not to be a widespread rule in parasitoid wasp venoms as suggested by the presence, in several species, of venom proteins that arose from gene duplications but whose key functional residues are often mutated [83,114]. This is in sharp contrast with PDVs whose genomes frequently contain gene sets forming large multigenic families and which may be co-expressed in host tissues [126]. The fact that the former are produced by the parasitic wasp itself and the latter at the expense of host insects may explain why diversification operated differently on both factors of virulence. Definitely, some like to be exhaustive at someone else’s expense.

PDV- and VLP-associated wasps are apparently undergoing a process of subfunctionalization, or functional partitioning, of their venom that probably started with the integrations of the ancestors of actual bracoviruses (BV), Ichnoviruses (IV) (the two subgroups of PDVs) and producers of VLPs in the genomes of different organisms belonging to Braconidae, Ichneumonidae and Figitidae [127]. In BV-associated parasitoids such as *C. inanitus* or *M. demolitor*, we observe that almost no overlap exists between venom proteins and PDV gene products [64,81] and, for *M. demolitor*, between venom and teratocytes [64]. According to Burke and Strand (2014) [64], this functional partitioning would provide “wasps the means to deliver and express effector molecules in hosts for protracted periods”. Consequently, the presence of the PDV allows relaxing the selective pressure exerted by a functional constraint identified by Fry *et al.* [110]: the need to produce a rapid effect in order to be effective and successful. Therefore it opens the possibility for these venoms to evolve in new directions, like the recruitment of venom proteins fulfilling structural functions or promoting cell growth and/or tissue differentiation. It seems to be the case for the venom of *C. inanitus* which contains an Imaginal disc Growth Factor (IDGF)-like protein (Ci-48b) and two putative mucin-like peritrophins (Ci-23c and Ci-220) [81].

Dorémus *et al.* (2013) [56] have suggested that several IV-associated wasps, such as *H. didymator*, have been further in the subfunctionalization process in producing venoms which reveal to be unnecessary to the success of the parasitoid although a number of venom proteins are still abundantly produced. Loss of regulatory functions may have followed viral acquisition or alternately, acquisition of these functions was only performed by the symbiotic virus. In parasitic wasps whose venom glands produce VLPs such as *L. herotoma* and *L. boulardi*, the most abundant venom proteins are in fact constitutive of the VLP capsid [69,114]. Since VLPs are devoid of nucleic acids and cannot externalize the production of regulatory proteins in parasitized host, venom gland plays here the role of “VLP factory”.

These examples highlight how, in parasitoids, venom composition and functional diversity are interdependently linked to the evolution of other factors of virulence. Here probably reside the main origins of their singularity regarding other animal venoms. Structural and functional constraints only explain a part of parasitoids’ venom complexity. Existence of inter-individual variability is another important parameter to understand how venom complexity and diversity have arisen.

#### 3.2.2. Populational and Ecological Factors

Studies led on BV-associated parasitoid wasps of the Microgastrinae complex have recently suggested that the highly diverse gene content of BV genomes could drive adaptation or specialization of parasitoid wasps to particular hosts [128]. For instance, former cross-protection experiments with species of the genus *Microplitis* have shown that BV-mediated immunosuppression was one important determinant of host range along with other factors [129]. Investigating similarly whether venom composition and effects could influence major life traits of parasitoid Hymenoptera, and could in turn be influenced by ecological constraints, necessitates the study of particular biological models. It can be achieved for example through the study of species devoid of symbiotic viruses and offering intraspecific variations of their venomous properties and successful parasitism rates (SPRs) toward a reference host model.

In the case of the evolution of the genus *Asobara* (Braconidae: Alysiinae), the cross-influences of the levels of resistance displayed by local species or strains of *Drosophila* hosts and of levels of virulence exhibited by sympatric parasitoid species are quite well documented [130]. The richness of these interactions has led to a surprising diversification of the composition and functional properties of the venomous secretions in *Asobara* parasitoids with direct and major consequences on the strategies of virulence of these species [116,131,132].

The SPR of the solitary endoparasitoid *Asobara tabida* towards *D. melanogaster* has been correlated with geographic localization [133]: the strain called A1 originates from the south of France and develops more successfully on *D. melanogaster* than the WOPV strain originating from the Netherlands [134]. The SPR of both strains in controlled conditions has been shown to be of 74% ± 2.6% and 18.8% ± 8.8%, respectively [42]. In this biological system, the parasitoid female lays a single egg into a young *Drosophila* larva, which may escape encapsulation if its chorion strongly adheres to the basal lamina surrounding the internal tissues of the host [135] (Moreau S., unpublished data). If an *A. tabida* egg is not able to bury itself between the host’s organs, it is rapidly encapsulated by circulating hemocytes, unless it has been oviposited into a host deprived of encapsulation abilities [133,136]. Whatever the outcome of the parasitic relationship, parasitized *D. melanogaster* larvae retain substantially their ability to mount effective immune reactions but experience a transient paralysis, an altered weight gain and a significant increase in the time required before the onset of pupariation [42,137]. These observations suggested that factors of parasitic origins, and notably venoms, had a precocious effect on activity and development of parasitized hosts, but almost not on their immunity, and that crucial difference could take place between the two strains studied. Interestingly, while parasitism by both strains induced equivalent mortality rates before the parasitoid’s emergence (approximately 20%), experimental injection of venom proteins from the WOPV strain significantly increased the mortality rate of *D. melanogaster* larvae. At the highest tested dose of venom (equivalent to a tenth of the venom produced during the first five days after emergence), 95% of *D. melanogaster* larvae died before reaching the pupal stage and none completed its development. In comparison, when the same quantity of venom proteins from the A1 strain was injected into *D. melanogaster* larvae, the observed mortality rate was only 35%. Venoms of both strains also exhibited variations in their ability to induce transient paralysis, the venom of the WOPV strain having the strongest effect [42]. Finally, electrophoretic profiles of venom extracts showed minor band differences (Moreau S., unpublished data). The abilities of *A. tabida*’s venom to induce host paralysis, to be lethal at high doses and most probably to delay development in parasitized hosts, are reminiscent of its ectoparasitic origins [138], even though its lifestyle is now undoubtedly endoparasitic. On the basis of their ancestral functional legacy and despite the loss (or the non-acquisition) of an immune-suppressive venom, geographically distant populations of *A. tabida* have thus evolved at least two strategies to adapt to endoparasitism. Eggs of the A1 strain take benefit from the increased stickiness of their chorion and from the lesser toxicity of their mother’s venom to successfully parasitize immune-reactive host larvae. Conversely, the eggs of the WOPV strain need to develop into immunocompromised hosts and the higher toxicity of the female’s venom could serve here to weaken or even eliminate some potential competitors (e.g., eggs of *L. boulardi* already present in the host) in order to allow a kleptoparasitic development of *A. tabida* eggs [133].

Countless other inter-individual variations in venom composition have occurred within hymenopteran parasitoids over the past millions of years of evolution and many of them have probably been selected under constraints imposed by interacting species within local communities. This question is now the subject of renewed interest [119,139] and should benefit from the availability of high-throughput sequencing methods that provide access to the inter-individual variability of venom gland gene expression within natural populations.

Beyond understanding the evolution of particular parasitoids-hosts relationships, investigation of the venom gland content may also directly inform us about the evolution of the order Hymenoptera. The venom composition of C. *inanitus* hence appeared as a mixture of conserved venom components and of recent proteins potentially specific of the lineage [81]. The phylogeny of several conserved venom proteins has been reconstructed and the authors identified Allergen 5 proteins, a group of major allergen components of ants and wasps venoms, as one of the most ancient family among insect venom proteins: the ancestral *Allergen 5* gene was likely already expressed by the venom glands of the common ancestor to Ichneumonoidea and Aculeata, 155 to 185 million years ago and has apparently been lost in Apoidea. This study has also confirmed that genes coding for honeybee’s Major Royal Jelly Proteins derived from a progenitor gene (*yellow-e3*) which probably possessed an ancestral venomous function, as previously suggested [140,141]. These examples show that aside from quantitative benefits expected from the achievement of extensive inventories, combined genomic, transcriptomic and proteomic approaches constitute appropriate tools to explore evolutionary purposes. Such approaches have notably been used with success to unravel the origins of PDVs produced by parasitoid wasps [62]; reconstructing the composition of some “paleovenoms” should thus become an achievable objective with exciting perspectives. Transcriptomic data can also help us understand the functioning of venom glands cells through the identification of gene products directly involved in production, delivery and activation of venom toxins of a broad range of venomous animals, such as the dipeptidyl peptidase IV (DPPIV) family enzymes [56].

## 4. Pharmaceutical and Biological Potential of Parasitoid Wasp Venoms

With a complexity generally comprised between 10 to 100 different venom proteins and peptides per species, the 250,000 known hymenopteran parasitoid species represent a source of millions of bioactive molecules which remain almost totally unexplored. The applied perspectives expected from their study are just as vast. In medical areas, they range for instance from the prevention and treatment of venom hypersensitivity to the discovery of innovative drug candidates. Given that parasitoid venoms also attract a growing attention as a rich source of bioactive substances for the control of insect pests [142], an advantage could be taken from the acquisition of a better knowledge on venom composition in a greater number of species, to optimize new strategies of integrated pest management.

### 4.1. Pharmaceutical Perspectives

The therapeutic value of venom immunotherapy to improve the quality of life of patients which are hypersensitive to the venom of social Hymenoptera is acknowledged since more than eighty years [143,144]. Recently, venomic approaches have allowed the discovery of new venom constituents which were proven to be of immunological significance and have opened the way to optimization of immunotherapeutic strategies through the use of cocktails of recombinant allergens [70]. Interestingly, the toxicity, allergenicity and algogenicity (potential for pain induction) of solitary predatory or parasitoid wasps towards human and domestic animals have almost never been assessed in laboratory. Although rare, accidental envenomation events have nevertheless been documented [105]. The continued increase in human populations’ densities could expose a greater number of people to these non-intentional contacts.

On the other hand, a number of venom serine proteases from wasps and bees were shown to exert a potent anticoagulant effect, inhibiting platelet aggregation and degrading the β-chain of fibrinogen [145,146]. Enzymes with similar functions have been reported from the venom of the ectoparasitoid *N. vitripennis* [70] and from those of the endoparasitoids *P. hypochondriaca* [99], *P. puparum* [113] and *A. japonica* [71]. Their study would raise potential applications for the treatment of thrombotic disorders.

Venoms of Hymenoptera also often contain antimicrobial peptides (AMPs) [32,147,148,149] or may stimulate the antimicrobial immune defenses of the targeted organism [105]. Such molecules may serve as templates to inspire new antibiotic agents, an invaluable resource considering the worrying current increase in the number of multi-drug resistant pathogens and the expected changes in the distribution of terrestrial ectotherms and epidemiology of infectious diseases which are likely to be induced by the ongoing global warming [150,151]. The antinociceptive effects of other venom peptides involved in the blockage of ionic channels can also represent potential sources for drug development to treat pain. Several examples of neurotoxic peptides from solitary and parasitoid wasps are already known [12,13,152].

Finally, one of the most interesting properties of venom components and venom cocktails are probably their natural stability as injectable solutes, their effectiveness in reaching targeted tissues and their ability to synergize their actions, shaped by millions years of “R&D”. Their features may inspire the design of recombinant hydrolases and innovative strategies of enzyme replacement therapy for patients suffering from rare lysosomal storage disorders [95,153]. Other venom molecules with protease inhibiting, pro-apoptotic or cytotoxic properties and described from several endoparasitoid species [94,95] may also worth to be considered for the development of anti-tumor or anti-viral agents.

### 4.2. Biological Control: Development, Reproduction and Immune Modulators

Twenty-one years ago, Zeneca Ltd. patented a synthetic DNA capable of expressing a venom peptide from *Conus* marine snails in pest insects via a baculoviral vector [154]. The year after, the same company patented a chimeric toxin resulting from the fusion of part of an endotoxin from *Bacillus thuringiensis* and an insecticidal venom toxin from the scorpion *Androctonus australis* Hector [155]. The chimeric toxin was thought to be applied directly to crop plants or to be produced by transgenic plants and delivered to pest insects through ingestion. In 1996, several patent applications concerning the potential use of *B. hebetor* venom neurotoxins as insecticidal toxins have been simultaneously deposited by Sandoz Ltd. [156], Zeneca Ltd. [157] and NPS Pharmaceuticals, Inc. [158,159]. The U.S. Patent by Quistad *et al.* (1996) [156] was notably directed to “toxins active against insects which are isolated from the parasitic wasp *B. hebetor*, the nucleic acids which encode the toxins, cloning of the toxins, use of the toxins to control insects, and genetically engineered virus vectors carrying the toxin gene”. Many have seen in these patent applications the opening of a new era in the use of parasitoid wasps in biological control, not only as living organisms, but also as sources of genes and molecules of interest to control pests [95,160,161]. However, because sequence information and experimental results were kept confidential, these patents have seriously hindered the diffusion of a useful knowledge. They have resulted in delaying both fundamental works about venom diversity and evolution, and the effective use of interesting molecules in the field. Beyond the fact that these “inventions” constitute regrettable attempts to enclose and usurp the genetic patrimony of wild species and data constitutive of the common knowledge of the human kind, they fuelled the public mistrust towards biotechnologies and the potential use of venom compounds from parasitoids for plant protection. In 1999, the British registered charity ActionAid was already wondering about the possible impacts on environment of genetically modified (GM) crops or baculoviruses expressing the *B. hebetor*’s venom toxins [162]: what would happen if the venom toxins were ingested by non-targeted organisms or if their genes were horizontally transferred to microorganisms or to wild plants? Are they allergenic or toxic to humans? Will these genetically modified organisms contribute to improving or degrading the situation of farmers in developing countries? It seems that answers to these important questions will also remain confidential for a while.

A very different approach has been followed during the next decade by a group working on the venom of the widely used biological control agent *Aphidius ervi* (Braconidae: Aphidiinae). The venom of *A. ervi* induces the castration of the pea aphid *Acyrthosiphon pisum* via the specific degeneration of the germaria and of the young apical embryos [54]. The sequence of the bioactive venom component inducing castration was published in 2007 [31]. It corresponds to a dimeric γ-glutamyl transpeptidase (γ-GT) which acts by specifically triggering apoptosis in germarial cells and cells of the ovariole sheath of the parasitized aphid. Recently this γ-GT has also been found in egg extracts of *A. ervi* which suggests that the expression of the venom enzyme would not be restricted to the venom gland of the parasitoid [163]. In addition these authors have precisely dosed the quantity of venom γ-GT injected in the aphid host during oviposition (approximately 4 ng) thanks to an elegant and transferable experimental setup relying on chitosan beads. Finally, a third group of investigators has recently performed a combined transcriptomic and proteomic approach on the venom of *A. ervi* [80]. Surprisingly, their work has revealed the presence of two additional γ-GTs in the venom, from which one would not be functional while the other would represent the product of an allelic variant of the original *γ-GT* gene. Interestingly, a reconstruction of the phylogenetic relationships between known hymenopteran γ-GTs suggests that an independent and converging duplication event would be at the origin of the presence of two other γ-GTs in the venom of the ectoparasitoid *N. vitripennis* [70,80]. Finally, the presence of an endoplasmin has also been detected in the venom of *A. ervi*. This protein, which belongs to a family of molecular chaperones could play a role in the transport and stabilization of the other venom proteins including the γ-GTs [80]. If it was confirmed, this finding could have important implications for future applications that would aim at using γ-GTs or other venom proteins to efficiently control aphid populations. This example illustrates, if we needed reminding, how open collaboration more than mercantile enclosures, encourages the acquisition of useful knowledge and makes progress possible.

The most advanced project to date concerns the selection of venom proteins from the endoparasitoid *P. hypochondriaca* able to help the control in the field of two pest insects, *Lacanobia oleracea* and *Mamestra brassicae* [164,165]. The originality of the envisaged strategy of control resides here in the use of the immunosuppressive properties of two venom proteins (VPr3 and VPr1) to increase sensitivity of pest insects to biological control agents (BCA) such as *Beauvaria bassiana* and *B. thuringiensis* (Richards and Dani, 2008). Injections of the recombinant rVPr1 suppressed the ability of *L. oleracea* and *M. brassicae* to mount hemocyte-mediated immune responses [165]. Two modes of delivery of rVPr1 to the targeted pest insects are studied in view of future practical applications: either by directly spraying rVPr1 onto plants attacked by the pests (which would require protecting the protein from degradation and inactivation) or via the expression of rVPr1 by the BCA itself [164]. The latest option would necessitate a careful development to avoid a reckless widening of the biological spectrum of BCAs, notably towards non-targeted species of Lepidoptera.

## 5. Conclusions

Despite the large diversity of parasitoid wasp species, there are only a small number of venom proteins that have been described from the wasps. There is a wealth of unexplored biomolecules present in parasitoid venoms that are of value in basic evolutionary studies, venom biology, host-parasite interactions, evolution of life strategies, and may potentially contain components that could be used in agriculture and pharmacology. The available state-of-the-art approaches in proteomics and transcriptomics provide us with valuable tools and unique opportunities to explore these diverse biomolecules. By characterizing parasitoids’ venoms at the functional level, we can gain a better understanding of neglected interactions to achieve knowledge that can enable us to utilize them for improved appreciation of life and diversity, pest management and health.
